# Human Bocavirus infection among children with respiratory tract infection in Ibadan, Nigeria

**DOI:** 10.1099/acmi.0.000356

**Published:** 2022-05-23

**Authors:** Olajumoke Olufunmilayo Joseph, Johnson Adekunle Adeniji, Adedayo Omotayo Faneye

**Affiliations:** ^1^​ Department of Virology, Faculty of Basic Medical Sciences, College of Medicine, University of Ibadan, Ibadan, Oyo, Nigeria

**Keywords:** Human Bocavirus 1, childhood respiratory tract infection, phylogenetic analysis, Ibadan

## Abstract

**Background.** Human Bocavirus (HBoV), which is an ssDNA virus of the family *Parvoviridae*, is responsible for 21.5 % of childhood respiratory tract infections (RTIs) annually. Among the four genotypes currently known, HBoV-1 has been associated with acute RTI. Although there have been studies on HBoV in some countries, there is limited information on this virus in sub-Saharan Africa where there is the highest burden of RTI. This study aimed to characterize the circulating strains of HBoV in Ibadan, Nigeria.

**Methods.** Nasopharyngeal and oropharyngeal swab samples were collected from 333 children ≤5 years old presenting with RTI attending hospitals in Ibadan, whose parents assented, from 2014 to 2015. Twenty-three HBoV isolates were sequenced after a nested PCR and phylogenetic analysis was carried out using mega 6 software.

**Results.:**

A total of 27 children tested positive for the HBoV-1 genotype by PCR and 23 of the 27 isolates were successfully sequenced. The 23 HBoV-1 isolates from this study have been assigned GenBank accession numbers KY701984–KY702006. Phylogram analysis indicated that the isolates belong to the same clades. Six isolates aligned closely to the reference strains ST1 and ST2, while 17 isolates showed a high level of divergence to the reference isolates.

**Conclusion.:**

This study highlights the contribution of HBoV to RTIs in Nigeria and that HBoV-1 strains are associated with the infection.

## Data Summary

**Fig. 1.**
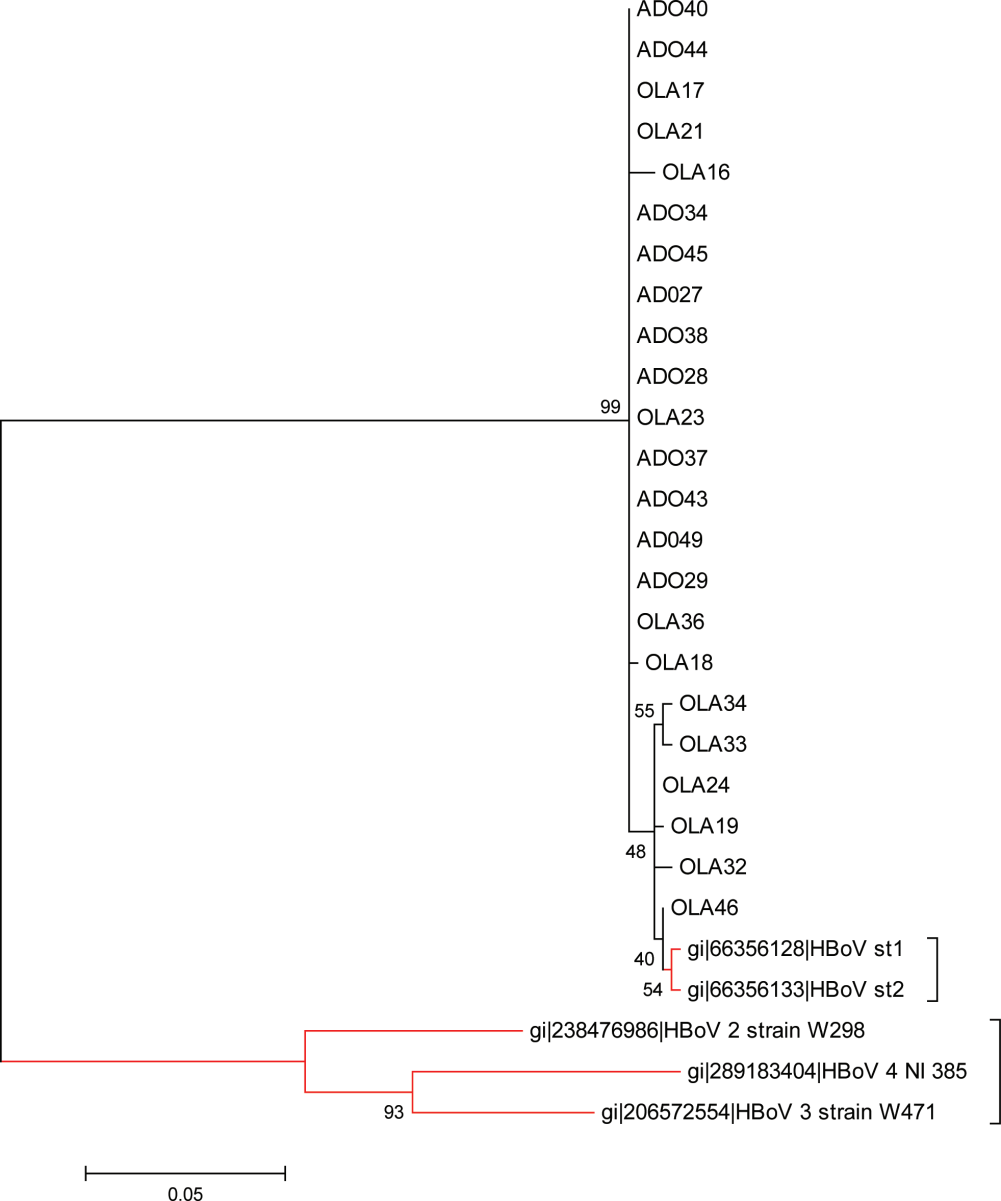
Phylogram of isolates of this study with reference strains. Phylogenetic tree with 1000 bootstrap replicates generated by neighbour-joining using the clustal w package in mega 6. HBoV1 isolates from this study are on black branches while the reference HBoV1 strains are on red branches. Scale bar refers to a phylogenetic distance of 0.05 nucleotide substitutions per site.

**Fig. 2.**
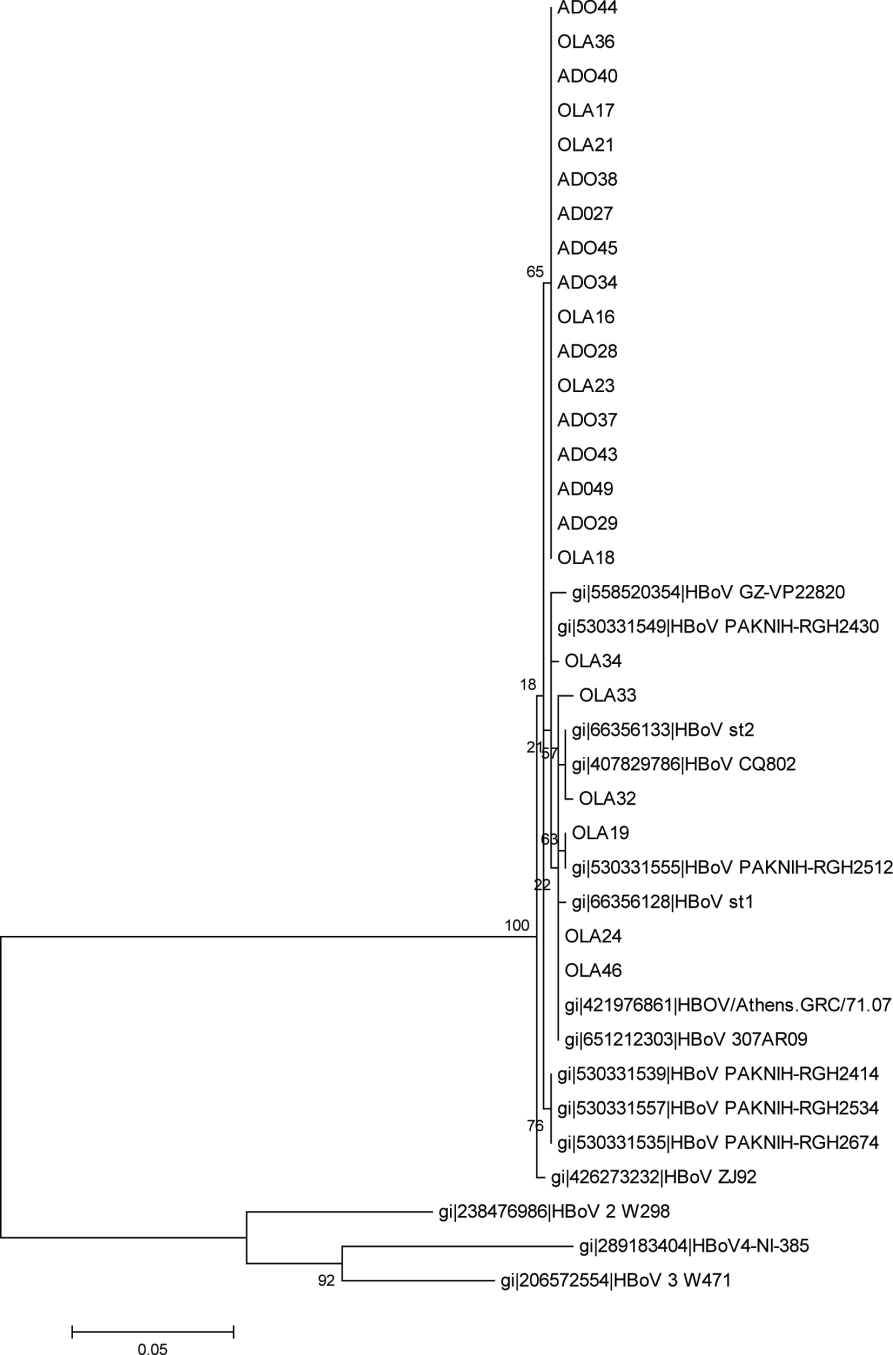
Phylogram of the study isolates with some other similar strains. Phylogenetic tree with 1000 bootstrap replicates generated by neighbour-joining using the clustal w package in mega 6. The HBoV1 isolates of this study were compared to some other similar strains from other studies. Scale bar refers to a phylogenetic distance of 0.05 nucleotide substitutions per site.

The HBoV1 isolates in this study have been assigned GenBank accession numbers KY701984–KY702006. Figures 1 and 2 are available on Figshare: https://doi.org/10.6084/m9.figshare.18681488 [1].

## Background

Respiratory tract infections (RTIs) are responsible for about 25 % of annual childhood deaths worldwide. Human Bocavirus (HBoV) has been linked with 21.5 % of childhood RTI annually [2]. HBoV1 has been associated with childhood RTI in both developed and developing countries, causing some severe cases of bronchopneumonia and bronchiolitis. Studies have shown that the virus is prevalent in children age <5 years, with the four genotypes often causing different clinical symptoms [3]. Also, since the discovery of the virus from nasopharyngeal aspirates in 2005 by Allander and colleagues, it has been observed that HBoV1 is the fourth most common virus in respiratory samples [4].

HBoV is a relatively newly identified DNA virus that belongs to the family *Parvoviridae* [5]. The virions are small, icosahedral and non-enveloped, with a negative sense and linear single stranded genome of ∼5.3 kb [6]. The HBoV genome codes for two non-structural proteins (NS1 and NP1) and two structural proteins (VP1 and VP2). The virus has been classified into four genotypes: HBoV-1, -2, -3 and -4, based on the nucleotide divergence of the VP1 capsid region [6]. HBoV-1 infection in children is often identified by wheezing, whereas HBoV2–4 has been found to be involved in childhood diarrhoea cases and acute flaccid paralysis [7].

Although studies have been carried out in some parts of Asia, Australia, Europe and America, there is still a dearth of information on circulating strains of the virus in sub-Saharan Africa, especially in Nigeria. However, due to the frequent detection of HBoV in co-infections with several other respiratory viruses, understanding the pathogenicity of the virus has remained a puzzle [8].

The present study aimed to characterize the circulating strains of HBoV among children ≤5 years old in Ibadan, Nigeria, presenting with symptoms of RTI.

## Methods

A total of 333 children ≤5 years old, showing signs of acute RTI, whose parents had given consent, were recruited for this study. Sterile swab sticks were used to collect oropharyngeal (OP) and nasopharyngeal (NP) samples from each child and transported immediately to the laboratory in sterile vials containing 2 ml of viral transport medium (VTM). Although the methods followed in this paper address a specific scientific problem, it is hard to include relevant controls in this study. DNA was extracted from the swab samples using a commercial DNA extraction kit by Jena Bioscience (Germany) according to the manufacturer’s instruction. HBoV was detected from the DNA in a nested PCR procedure as described by Kapoor and colleagues [9] using primers targeting the VP1/2 region of the virus genome (Table 1) and red Load Taq Master kit by Jena Bioscience in a 25 µl reaction. The first round of cycling comprised 95 °C for 2 min, 10 cycles at 95 °C for 35 s, 58 °C for 60 s and 72 °C for 60 s (with a decrease of 0.5 °C in annealing temperature after each cycle), 30 cycles at 95 °C for 30 s, 54 °C for 45 s and 72 °C for 45 s, and a final extension of 72 °C for 10 min. The second round had similar conditions to the first, with a difference in the initial annealing temperatures, 60 °C in the first group of cycles and 58 °C in the second [9].

**Table 1.** Oligonucleotide primers used for the amplification and sequencing of HBoV

Primer	Sequence, 5′–3′	Sense	Reference
HBoV 1F	CGCCGTGGCTCCTGCTCT	+	[9]
HBoV 1R	TGTTCGCCATCACAA AAGATGTG	−	
HBoV 2F	GGCTCCTGCTCTAGGAAATAA AGAG	+	
HBoV 2R	CCTGCTGTTAGGTCGTTGTTGTATGT	−	

All samples that were positive for HBoV (showing the expected 575 bp) at the end of the second round of the nested PCR were purified and sequenced. The query DNA sequence generated from the 23 HBoV isolates was extracted in Fasta format and manually edited using mega 6 software [9]. Similar sequences to the HBoV sequences in GenBank were checked using the blast (Basic Local Alignment Search Tool) program in the National Center for Biotechnology Information (NCBI) database. WHO HBoV reference sequences were downloaded from NCBI and a multiple alignment with the sequences generated was carried out using mega 6 software. Phylogenetic analysis was performed by neighbour-joining using the cluster w package in mega 6 and maximum-likelihood trees were generated after correcting for multiple substitutions, complete removal of positions that contained gaps and estimating reliability based on 1000 bootstraps. Multiple alignment and phylogenetic analysis of the sample sequences with previous sequences from Nigeria and worldwide was also done.

The nucleic acid sequences generated from the samples was translated into amino acid sequences from the ORFs within each sequence using mega 6 software. The alignment of amino acid sequences of the 23 HBoV isolates was compared with those of the WHO reference strains, Nigerian and other similar strains from around the world. Maximum-likelihood trees were generated after correcting for multiple substitutions, complete removal of positions that contained gaps and estimated reliability based on 1000 bootstraps.

Data entry, cleaning and data analysis were performed using SPSS statistical software, and descriptive statistics were presented using tables, graphs and charts.

## Results

A total of 333 children aged 5 years and below presenting with cough, wheeze, breathlessness, fever, nasal congestion, catarrh, vomiting, tonsillitis, otitis media, rash, diarrhoea, lack of appetite, bronchiolitis and bronchopneumonia were recruited from September 2014 to August 2015 from hospitals and health centres in Ibadan, Oyo State, Nigeria. In total, 170 of the children were male and 155 females. Table 2 shows details of the study population.

**Table 2.** Characteristics of the study population

Variable	Category	Frequency	Gender		Total positive HBoV	Percentage	sd	*P* value
			Male/no. positive	Female/no. positive				
Age	0–6 months	133	68/0	65/2	2	39.9	1.636	0.001
	>6 months − 1 year	51	28/1	23/1	2	15.3		
	>1–2 years	67	37/8	30/3	11	20.1		
	>2–3 years	27	12/0	15/3	3	8.1		
	>3–4 years	29	19/2	10/3	5	8.7		
	>4–5 years	26	14/0	12/4	4	7.8		
Total		333	178/11	155/16	27			

Twenty-seven (8.1 %) of the 333 children tested were positive for HBoV infection, 16 (59.3 %) of whom were female. The age group ˃1–2 years showed the highest prevalence. Twenty-three of the total HBoV-positive isolates were sequenced and characterized (Tables 2–4). All the isolates were of HBoV1 genotype. The maximum-likelihood tree and the pairwise distance showed that 17 (out of 23) isolates were highly divergent from the others, although all the 23 isolates were HBoV1 (Figs 1 and 2).

**Table 3.** Frequency of symptoms among HBoV-positive children

Symptom	Frequency
Cough	27
Wheeze	7
Breathlessness	5
Fever	9
Nasal congestion	16
Catarrh	27
Vomiting	3
Itches	0
Tonsillitis	1
Diarrhoea	1
Otitis media	2
Rash	2
Anorexia	7
Restlessness	0

**Table 4.** List of isolates with description of their source

Sample ID	Gender	Age	Symptoms
OLA17	F	4 years	Cough, nasal congestion, catarrh
ADO40	F	3 years	Cough, wheeze, catarrh
OLA21	F	3 years	Cough, nasal congestion, catarrh, anorexia
ADO38	F	2 years	Cough, wheeze, catarrh
ADO27	F	1 year	Cough, wheeze, breathlessness, nasal congestion, catarrh
ADO45	F	5 years	Cough, catarrh
ADO34	F	4 years	Cough, wheeze, catarrh
OLA16	M	1 year	Cough, fever, nasal congestion, catarrh, anorexia
OLA18	F	1 year	Cough, nasal congestion, catarrh, otitis
ADO28	F	2 years	Cough, catarrh
OLA23	F	2 months	Cough, fever, nasal congestion, catarrh
ADO37	M	3 years	Cough, wheeze, catarrh
ADO43	F	3 years	Cough, catarrh
ADO49	M	5 years	Cough, wheeze, catarrh
ADO29	F	2 years	Cough, wheeze, catarrh
ADO44	M	3 years	Cough, wheeze, catarrh
OLA36	M	1 year	Cough, fever, Nasal congestion, anorexia
OLA34	F	4 years	Cough, nasal congestion, catarrh, vomiting
OLA33	F	1 year	Cough, nasal congestion, catarrh, tonsillitis, anorexia
OLA24	M	1 year	Cough, fever, nasal congestion, catarrh, vomiting
OLA32	M	9 months	Cough, breathlessness, fever, nasal congestion, catarrh
OLA46	F	1 year	Cough, breathlessness, fever, nasal congestion, catarrh, anorexia
OLA19	M	1 year	Cough, breathlessness, fever, nasal congestion, catarrh

The phylogenetic tree reconstructed with the sequenced isolates and WHO reference strains using mega6 [10] showed that the 23 isolates of this study clustered with HBoV1 WHO reference strains, although 17 of the isolates showed a higher divergence (Figs 1 and 2). The divergence of the sequences of the study isolates and the reference strains sequences revealed an overall mean distance of 0.0 within the isolates and 0.1 when compared with WHO reference strains.

## Discussion

In this study, HBoV was detected in 8.1 % of the 333 children recruited. The detection rate observed in this study is higher than the 6.8 % reported by Moreno *et al*. [11] in Argentina, 7.2 % reported by Tran *et al.* [12] in Vietnam and much higher than the 1.2 % reported by Niang *et al* [13] in Senegal, but lower than the 16.8 % reported by [14] among Kenyan children. This difference could be as a result of sampling strategy, severity of illness in the children recruited and different climates. Studies have revealed that HBoV infection among children 0–5 years old seems to be higher in cold and rainy seasons, and it has been shown that the virus is involved in RTIs of children yearround, globally. At the time of this study, to the best of our knowledge, there was a paucity of information on HBoV infections among children in Nigeria. Although not significant, females (59.3%) were found to have a higher prevalence of HBoV in this study (Table 2). This is similar to what was previously reported in India by P. Bharaj, *et al.* [15] and in Saudi-Arabia by Abdel-Moneim, *et al*. [16], but different from reports by Symekher *et al*. [14] among Kenyan children and Salmon-Mulanovich *et al*. [17] among children in South America, whose findings showed a slightly higher prevalence among male children. Children >1–2 years of age had the highest detection rate and showed symptoms of the highest severity (Table 2). This is similar to the findings of Abdel-Moneim *et al.* [16], whose study showed similar findings among children. Further studies on this virus are required globally to provide more information on its prevalence among children. Among the symptoms observed in children with HBoV in this study, cough (100%) and catarrh (100%) had the highest frequency, whereas vomiting (11%), tonsillitis (3.7%) and diarrhoea (3.7%) were the least observed symptoms (Table 3). The symptoms observed in children positive for HBoV infection in this study are similar to the reports of Hengst *et al.* [18], Korner *et al*. (2011) [19], Halise *et al*. (2016) [20] and Moesker *et al*. [21]. This suggests that laboratory screening for HBoV should be included in the hospital management of children ≤5 years old presenting with cough, catarrh and other respiratory tract symptoms in Nigeria, in order to provide information on he appropriate course of treatment. In addition, paediatricians need to be well informed on HBoV infection when advising parents or guardians who are involved in home-based care of children showing symptoms of RTI.

Of the 27 HBoV isolates detected in this study, 23 were successfully sequenced. The phylogram generated from the nucleotide sequence of the 23 HBoV isolates in this study and that of HBoV reference strains showed all the isolates clustering with the reference strain of HBoV-1 (Figs 1 and 2). This is similar to the findings of Johanna *et al*. (2014) [22], Abdel-Moneim *et al.* [16] and Principi *et al.*, [23], who reported HBoV-1 as the genotype mostly found associated with respiratory distress in children.

Six of the 23 isolates in this study showed a very high level of similarity (40–55 %) with ST1 and ST2, which are the WHO reference strains for the HBoV-1 genotype [5] while the remaining 17 isolates showed lower similarity to the reference strains. The six isolates that clustered most closely to the reference strains for HBoV-1 (Figs 1 and 2) were from children who exhibited symptoms of high severity (Tables 3 and 4). This is similar to the findings of Sebastien *et al*. (2017) [24] and Vasiliki *et al*. (2013) [25], whose study indicated that HBoV-1 isolates aligning with the HBoV-1 reference strains are often obtained from children showing symptoms of severe acute RTIs. This could be because the HBoV-1 reference strains were isolated from children with severe acute respiratory tract illness by Allander *et al.* [5]. The remaining 17 isolates that showed a higher divergence to the reference strains were from children who exhibited less severe symptoms of respiratory tract illness (Table 4). This corroborates the reports of Kapoor *et al.* [9] and Abdel-Moneim *et al.* [16], which showed that HBoV-1 was detected from both severely ill children and those presenting with mild respiratory symptoms.

There was low level of diversity in the nucleotide and amino acid differences within the HBoV-1 isolates of this study (i.e. an overall mean distance of 0.0 within the isolates and 0.01 when aligned to the WHO reference strain sequences). This shows the high level of genetic homogeneity exhibited by the HBoV1 isolates of this study, and supports the findings of Allander *et al.* [5], Arthur *et al.* [8], Cheng *et al.* [6] and Ghietto *et al*. [26]. When comparing the isolates obtained in this study to other isolates in GenBank it was observed that majority of the isolates were closely related to those from Pakistan. This supports the findings of Kantola *et al.* [3] and Salmón-Mulanovich *et al*. (2010) [17], which suggests that there is frequent importation of foreign strains of the HBoV-1 genotype, and indicates that tourism plays an important role in transmission of the virus [3].

Finally, the 8.1 % prevalence of HBoV-1 that was found in this study showed that this virus strain is involved in childhood respiratory tract illnesses in Ibadan and is therefore of public health importance in Nigeria. Although the greatest number of HBoV-1 isolates (11) was obtained in January (Table 5), the short duration of the study prevents proper observation of seasonal patterns of HBoV-1 infection among children. Further investigation is needed to better understand the rate of transmission and symptoms in different seasons.

**Table 5.** Seasonal distribution of HBoV

Month of sample collection	HBoV positive		Total	*P* value
	Yes	No		
January	11	29	40	0.000
February	2	23	25	
May	2	7	9	
June	0	27	27	
July	0	17	17	
August	2	80	82	
September	1	79	80	
October	0	6	6	
November	1	6	7	
December	8	32	40	
Total	27	306	333	

## Conclusion

RTIs are implicated in a large number of childhood deaths globally, and HBoV-1 has been shown to be involved in the respiratory illness of children ≤5 years old. The findings of this study show that HBoV-1 is endemic in Ibadan and might be actively circulating among children in Nigeria. Also, there is a likelihood that the movement of people fromone country to another plays an active role in the transmission and evolution of the virus. This study therefore shows the need for continuous surveillance of HBoV and laboratory investigation of the role it plays in the disease process. It is also important to promote good hygienic practices in Nigeria.
